# Development of *p*-Coumaric Acid Analysis in Human Plasma and Its Clinical Application to PK/PD Study

**DOI:** 10.3390/jcm10010108

**Published:** 2020-12-30

**Authors:** Hohyun Kim, Yunkyoung Choi, Yongwun An, Young-Rim Jung, Jin-Yong Lee, Hong-Jin Lee, Jihoon Jeong, Zisoo Kim, Kyeongsoon Kim

**Affiliations:** 1 Korea Medicine Research Institute, Inc., Seongnam 13201, Korea; ykchoi@kmri.co.kr (Y.C.); ywan@kmri.co.kr (Y.A.); 2 Korean Medicine Hospital, Seoul 02447, Korea; jylee@kyh.ac.kr; 3 KyungHee University, Seoul 02447, Korea; 4 Department of Pediatrics and Adolescent Medicine, Hallym University Hospital, Chuncheon 24253, Korea; hjlee@hallym.ac.kr; 5 Department of Global Innovative Drugs, Graduate School of Chung-Ang University, Seoul 06974, Korea; Jhjeong3@cau.ac.kr; 6 Department of Pharmacology, College of Medicine, Chung-Ang University, Seoul 06974, Korea; ziskim@cau.ac.kr; 7 Department of Pharmaceutical Engineering, Inje University, Gimhae 50834, Korea; kskim@inje.ac.kr

**Keywords:** *p*-Coumaric acid, *Bambusae Caulis* in Taeniam, IGF-1, Osteocalcin, growth hormone therapy

## Abstract

It has been recognized that *p*-Coumaric acid (*p*-CA) has protective effects as an antioxidant, anti-inflammatory agent. A sensitive and efficient Liquid Chromatography-Mass Spectrometry (LC-MS) method for maximum determination of *p*-CA in human plasma has been established using Ultra-performance liquid Chromatography-tandem mass Spectrometry (UPLC-MS/MS). This study provides the developed analysis of *p*-CA extracted from *Bambusae Caulis* in Taeniam (BC) to examine the improvement of the treatment *p*-CA, IGF-1 and Osteocalcin level in human children which are important factors on the growth of children’s height through Pharmacokinetics/Pharmacodynamics (PK/PD) model. *p*-CA and internal standard in a plasma sample were detected by the Multiple Reaction Monitoring (MRM) scan mode with positive ion detection. The sample participating in the study was made of 34 subjects (placebo = 18, treatment = 16). The subjects were enrolled to be randomized to the control group and BC group. Randomized subjects took tested treatment twice a day, three capsules with oral administration (258 mg/capsule) each time after a meal. Standard calibration curves (reproducibility) were constructed and the lower limit of quantitation (LLOQ) for *p*-CA was found to be 0.2 ng/mL on injection of the sample into the UPLC-MS/MS system. Accuracy and precision were evaluated and the intra-accuracy was 99.2–103.8% with precision of 1.0–5.6% and inter-accuracy was 99.6–108.4% and precision of 1.3–6.4% for *p*-CA. The method has been successfully applied to PK/PD studies of *p*-CA in human plasma. The *p*-CA, BC in Taeniam extract increased the level of IGF-1 and Osteocalcin, and changed the height from baseline, which suggested that the *p*-CA could play an important role in longitudinal bone growth. Therefore, the *p*-CA extracted from BC in Taeniam might be a good alternative medicine to growth hormone (GH) therapy.

## 1. Introduction

Growth hormone (GH) therapy is a conventional treatment used for children with GH deficiencies [1,2]. Even though GH treatment is well known and is effective at accelerating longitudinal bone growth [3], it has several side effects which need medical attention [1]. It can set off many problems such as inhibiting protein degradation in many diseases and increasing insulin and IGF-1 levels [1]. GH treatment for children requires being typically administered daily and long-term until the child reaches adult height. Ultimately, GH long-term treatment can cause unusual increased weight, muscle, and abnormal lipids [4]. Furthermore, there are contraindicated conditions which should not use GH therapy such as tumors and in children and adolescents with closed growth plates. Other side effects can include headache, visual problems, nausea and vomiting, fluid retention (peripheral edema), arthralgia, myalgia, paresthesia, antibody formation, hypothyroidism and reactions at the injection site [4]. An additional apprehension related to GH treatment is its high cost, which most parents experience significant financial strain [4]. Hence, developing effective herbal medicines for bone growth is a quite noticeable alternative treatment with decreased side effects as well as finical burden. 

*Bambusae Caulis* in Taeniam (BC), a medicinal herb originating from the inner bark of Phyllostachys nigra var. henosis (Milford) Rendle (Poaceae), has shown many pharmacologic activities [5,6].

BC has been used as a traditional Chinese medicine for the treatment of hypertension and cardiovascular disease in China and Korea [5]. Additionally, BC has been recorded to relieve fever, vomiting, stomachache, diarrhea, and chest diaphragm inflammation in the material media of past dynasties in Chinese history, and has been certificated as a functional food material by the Ministry of Health in China [7,8]. *p*-Coumaric acid (*p*-CA), also known as 4-hydroxycinnamic acid, is a phenolic acid, which has been widely studied due to its beneficial effects against several diseases and its wide distribution in the plant kingdom [9]. *p*-CA is a common compound found in medicinal herbs, including BC. It has been used to treat various diseases in China and Korea [6]. Many researchers have explained the versatile medicinal activities of *p*-CA, including antioxidant, cardioprotective, antimelanogenic, antimutagenic, antiplatelet, anti-inflammatory, and immunomodulatory actions [10,11,12,13].

However, there have been still few studies with regard to the pharmacokinetics and pharmacodynamics of *p*-CA extracted from BC. Pharmacokinetics are essential to know how the drug administered to a living body is distributed, absorbed, metabolized, and excreted in the body. Whereas, pharmacodynamics is essential to clarify the quantitative effects and mechanisms of action of the drug on the human body and to enable guidance for practical application. There have been several reports concerning the *p*-CA in human and rat plasma samples using the Ultra-performance liquid Chromatography-tandem mass Spectrometry (UPLC-MS/MS) method. However, the effect of *p*-CA on bone growth has not yet been reported.

In the present study, a selective and sensitive LC-MS/MS method for quantitation of *p*-CA extracted from BC was used, and the pharmacokinetics of *p*-CA in human plasma was investigated. Moreover, we investigated whether the BC extract (*p*-CA) has the ability to improve bone growth in the children where the hypo-nephrosis with below 5% of the growth chart. We also estimated whether the BC extract (*p*-CA) can change total serum osteocalcin and IGF-1 levels, which are significant parameters for bone growth.

## 2. Materials and Methods

### 2.1. Analysis of p-Coumaric Acid and Pharmacokinetic Application

#### 2.1.1. Chemicals and Reagents

**Figure 1:**
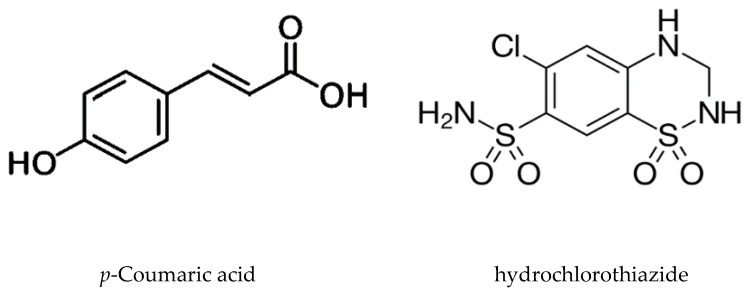
Chemical structure of *p*-Coumaric acid and hydrochlorothiazide.

*p*-CA (Figure 1) and hydrochlorothiazide were purchased from Sigma-Aldrich (Burlington, MA, USA). HPLC-grade acetonitrile and methanol, dichloromethane, methyl-t-butyl ether were purchased from SK chemicals (Ulsan, South Korea), and formic acid from Sigma-Aldrich (USA). A stock solution of *p*-CA (1 mg/mL) and hydrochlorothiazide (1 mg/mL) were prepared in the methanol. From these stock solutions, working standard solutions containing from 2 to 200 ng/mL *p*-CA were prepared by sequential dilution with the 50% methanol. *p*-CA (2, 10, 20, 50, 100 and 200 ng/mL) were prepared by spiking the working standard solutions into a pool of drug-free human plasma.

#### 2.1.2. Preparation of Standards

Stock solutions of *p*-CA were prepared in ethanol at concentrations of 1.0 mg/mL and then stored at −20 °C or below. The working solutions were prepared by diluting the stock solutions with water. Respective standard plasma to 200 μL was prepared by diluting 10% of the working solution with 90% of the frozen blank plasma under −70 °C, which was with a concertation of *p*-CA at of 0.2, 1, 2, 5, 10 and 20 ng/mL, and the standard plasms were transferred into microtubes, respectively.

#### 2.1.3. Sample Preparation

A 200 µL aliquot of human plasma sample was mixed with 10 µL of 500 µg/mL internal standard solution. To this, 200 µL of water was added after vortex mixing for 5 s, and then it was centrifuged at 13,000 rpm for 10 s. The sample mixture was loaded with Strata X-A 33µ polymeric strong anion 30 mg/1 mL SPE cartridge that was pre-conditioned with 1.0 mL methanol followed by 1.0 mL of water. The extraction cartridge was washed with 2.0 mL water and then followed by 1.0 mL of methanol. Analyte and IS were eluted with 2% formic acid in methanol and evaporated at 50 °C under a gentle stream of nitrogen. The dried extract was reconstituted with 100 µL mixture (80: 20, *v*/*v*) of 0.1% formic acid and acetonitrile. Aliquotd of 5 µL of the extract were injected into the UPLC-MS/MS system.

#### 2.1.4. UPLC-MS/MS Conditions

Tandem mass spectrometry was performed with a Xevo TQ-MS triple quadruple mass spectrometer (Waters Co., Manchester, UK) equipped with an electrospray ion source. The sample (5 µL) was delivered into the ESI source by UPLC (Model Acquity UPLC, Waters Co., Milford, MA, USA) with HECTOR-A C18 HPLC column (2.1 × 50 mm, 3 µm particle size). The mobile phase including 0.1% formic acid (solvent A) and acetonitrile (solvent B) in a gradient proportion was prepared to separate the analyte from the endogenous components. The gradient program was run from 20% B to 70% B in 1.3 min and ramped up to wash 80% B within 0.1 min and held for 0.6 min and down to initial conditions within 0.5 min and stayed there for 0.6 min. The flow rate was 250 µL/min and the total run time was 3.0 min.

The electrospray probe with nitrogen as the desolvation gas had a flow rate of 800 L/h. The desolvation probe temperature was 500 °C. 

Argon was used as a collision gas. *p*-CA and the internal standard were analyzed by the MRM scan mode with positive ion detection; the parameter settings were—capillary voltage at 3.0 kV, cone voltage at 20 V, extractor at 3 V, source temperature at 150 °C, collision energy at 10 eV, multiplier at 529 V, and dwell time of 0.20 s. Mass calibration was carried out by infusion of a calibration solution into the ion spray source. The peak widths of precursor and product ions were maintained at ∼0.7 mass unit at half-height in the MRM mode. 

#### 2.1.5. Pharmacokinetic Assay

In order to determine *p*-CA, in human plasma after oral administration (258 mg/capsule) of BC, a pharmacokinetic study has been performed with the developed analysis methods. Blood samples were collected from a vein before dosing and at 0, 0.25, 0.5, 0.75, 1, 1.5, 2, 3, 4, 6, 8, 10 and 12 h after dosing of *p*-CA.

### 2.2. Pilot Study with p-Coumaric Acid for Pharmacodynamic Application Study Population

All subjects were provided with informed consent before the initiation of the study procedures, checked it out and voluntarily agreed to take a part in this study. This study was conducted in accordance with the Declaration of Helsinki and International Conference on Harmonization Guideline for Good Clinical Practice. The protocol and informed consent forms were approved by the institutional review board of the Hanlim University (Chuncheon, Korea) and KyungHee University Oriental Medicine Hospital (Seoul).

All participating subjects were considered eligible for the study if the inclusion and exclusion criteria were met. Participants were immediately removed from the study if the investigator raised safety issues with the assessment.

#### Randomization and Experimental Procedure 

**Figure 2:**
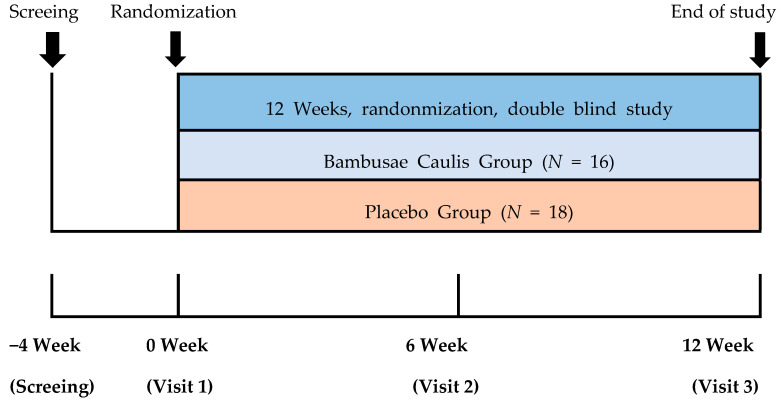
Subject visit flow. A total of 12 weeks, randomization, double blinded, placebo study Bambusae Caulis group (*n* = 16) and Placebo group (*n* = 18).

This study was conducted in a double-blind manner. The subjects who had written an informed consent form were screened according to screening test and then only eligible subjects were enrolled to be randomized to the BC group and placebo group after meeting all inclusion and exclusion criteria through evaluation from screening and scheduled visit. Randomized subjects took IP (investigational product) twice a day and visited the hospital every week in order to check vital, compliance, medical assessment, adverse events as well as other tests which were planned according to protocol at every visit (Figure 2). 

All subjects who completed all visits were followed up for safety observation. The randomized subjects to the BC group and placebo group took the IP twice a day, each time 3 capsules in oral administration (258 mg/capsule) after a meal.

### 2.3. Statistical Analysis 

The general characteristics of the study subjects were summarized by frequency and percentage for categorical data, and the result for continuous data was calculated as mean ± standard deviation. In order to test the difference between categorical variables between the two groups, a chi-square test or Fisher’s exact test was used. Testing the difference between two groups of continuous variables, an independent *t*-test was used when the data followed a normal distribution; otherwise, a Mann–Whitney’s U test was used. Shapiro–Wilk’s test was used to test the normality of continuous variables. SPSS 22 version (IBM Corp, Armonk, NY, USA) was used for all statistical analysis, and two-sided tests were used under a significance level of 0.05.

## 3. Results

### 3.1. Analysis of p-Coumaric Acid

#### 3.1.1. Specificity

**Figure 3:**
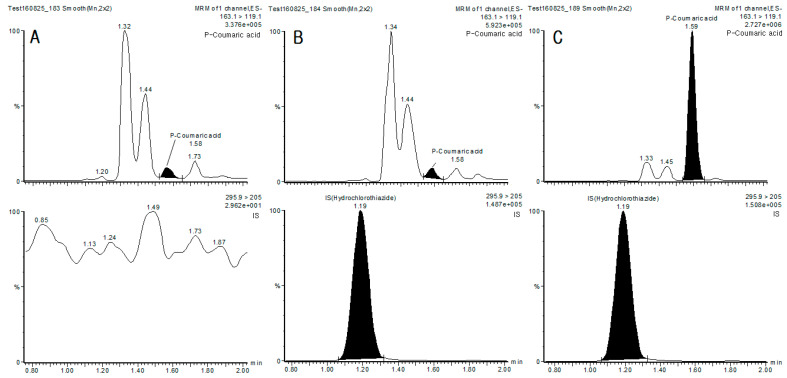
Representative Chromatogram of *p*-Coumaric acid (**top**) and internal standard (**bottom**) in human plasma. (**A**) Chromatogram profile of a blank plasma spiked with 500 µg/mL internal standard, (**B**) Chromatogram profile of plasma sample spiked with *p*-CA 0.2 ng/mL (LLOQ) and 500 µg/mL internal standard, (**C**) Chromatogram profile of plasma sample spiked with *p*-CA 20 ng/mL (ULOQ) and 500 µg/mL internal standard.

Specificity was evaluated by analyzing matrix blanks from six unique lots of matrix. Each blank sample was tested for interference, and specificity was confirmed by the absence of any peak > 20% of the mean peak response of the lower limit of quantification (LLOQ) in the intra-batch run. Typical chromatograms of blank plasma, spiked plasma sample are shown in Figure 3.

#### 3.1.2. Matrix Effect and Recovery

The matrix effects for *p*-CA in human plasma were 95.72 ± 6.6% and 90.07 ± 3.0% (*n* = 6 for each concentration level) at two QC concentration levels of 0.6 and 16 ng/mL, respectively. The extraction recoveries for *p*-CA in human plasma were 57.0% and 47.4% (*n* = 3 for each concentration level) at two QC concentration levels of 0.6 and 16 ng/mL, respectively. Mean recovery for hydrochlorothiazide (500 µg/mL) as the internal standard was 60.2% (*n* = 3 for each concentration level).

#### 3.1.3. Accuracy and Precision

Accuracy and precision were evaluated from five replicates of QC samples at four concentrations of 0.2, 0.6, 8 and 16 ng/mL. Intra-accuracy and precision were determined by analysis of at the least of five replicates at each QC level within a day. Inter-accuracy and precision were determined by analysis of at the least of three replicates at each QC level within five days. The intra-accuracy was 99.2–103.8% with precision of 1.0–5.6% and the inter-accuracy was 99.6–108.4% with precision of 1.3–6.4% for *p*-CA (Table 1).

#### 3.1.4. Sensitivity and Calibration Curve

Standard calibration curves (reproducibility) were constructed on different working days (5 days) using the human plasma. The response was linear throughout the concentration range of the study, with the coefficient of determination (*r*^2^) always >0.9993. The correlation equation was y = 6819.89x + 1013.91 in human plasma. On the basis of a signal-to-noise ratio (S/N) of 10, the lower limit of quantitation (LLOQ) for *p*-CA was found to be 0.2 ng/mL on injection of the sample into the UPLC–MS/MS system. The LLOQ is defined as the lowest concentration of the analyte that can be measured with a coefficient of variation and accuracy both ±20%. These LLOQ values were sufficient for pharmacokinetic studies.

#### 3.1.5. Pharmacokinetic Application

**Figure 4:**
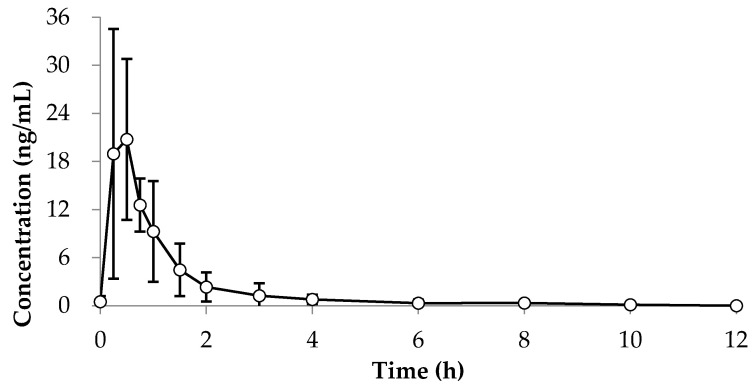
Plasma concentration-time curves for *p*-Coumaric acid in human plasma after oral administration of BC.

The developed method has been successfully applied for the pharmacokinetic study of *p*-CA in human plasma after oral administration of *p*-CA extracted from BC. The concentration-time curves are shown (Figure 4) and the corresponding pharmacokinetic data are shown in Table 2.

The described analytical method was used to analyze *p*-CA in human plasma samples after oral administration of BC. *p*-CA was absorbed rapidly and reached a maximum concentration (C_max_) and T_max_ of 21.95 ± 11.36 ng/mL and 0.50 ± 0.35 h, respectively. The T_1/2_ of *p*-CA was 0.9 ± 0.5 h. The AUC_t_ and AUC_inf_ of *p*-CA after oral administration of BC was obtained as 20.55 ± 1.50 and 20.82 ± 1.63 ng·h/mL, respectively. AUC_t_/AUC_inf_ was 98.7 ± 0.5%, which was found to be sufficient time to measure the parameters.

### 3.2. Pilot Study of Bambusae Caulis in Taeniam

The result of pilot study was conducted with the children whose growth was slow at below 5% of growth chart. The demographic data of the participants in this study are shown in Table 3. The enrolled participants were 16 and 18 for placebo and tested treatment, respectively. The number of the enrollments of this study was fewer than the sample size we calculated because this study was defined as a pilot study for the pivotal study with a large number of participants.

Table 4 indicates the alteration of height, IGF-1 and osteocalcin after placebo and tested treatment. The height difference after 12 weeks between both groups was evident, but not statistically significant. The checked IGF-1 and osteocalcin level are important factors on the growth of children’s height. The increase in IGF-1 and osteocalcin with tested treatment was observed and significant. In the placebo group, the significant decrease in osteocalcin was shown in contrast to that in the treatment group. 

The safety evaluation was assessed at each visit for the subjects who were administered with placebo and the tested treatment and evaluated for the primary endpoint. The evaluated safety parameters included physical examinations, hematological diagnostic tests and vital signs (Table 5). There were no unusual findings for all the participants. Considering the tested treatment has been used as a health supplement in China for a long time, it has been considered that there would be no problem in safety for children in Korea.

### 3.3. Sample Size Calculation

Sample size was calculated by using the test between two groups for the differences in height in visit 1 and visit 3 as effect size. The following results calculated 98 subjects for the case where the normality was satisfied, with the significance level of 0.05 being two-sided. The power was 90% and the effect size was 0.0666. Considering a 10% drop out, 110 subjects were needed as 98/0.9.

The interest in children’s height growth is growing more and more as the recent physical condition gradually becomes Westernized. In particular, it is of great interest that the physical development of low-growth children is also involved in social adaptation as well as educational and mental development. There have been a few investigations on herbal application in growth delay. Of them, effects of aqueous extract of Phyllostachyos (*Bambusae)* Caulis in Taeniam on longitudinal bone growth in adolescent rats was published, therefore there may be a possibility of development of BC as a dietary supplement or medical treatment for growth delay in children.

BC is a traditional Chinese medicine composed of about 50 kinds of caffeic acid, ferulic acid and *p*-CA [6]. It has been widely used as a Chinese medicine to treat diseases such as hypertension and cardiovascular disease in China and Korea [5]. Recently, in China and Japan, as a dietary supplement, many people enjoy drinking tea for psychological stability, and in Korea, herbal medicines are used as medicines to treat abdominal pain. We developed a new analytical method to validate assessment of *p*-CA as an indicator substance for quality and quantity control of BC extract. 

In this study, an analysis method using *p*-CA present in BC extract as an indicator was developed using UPLC-MS/MS. According to the results of this study, *p*-CA can play a role as a large amount of indicator substance is present in BC extract, and by developing its analysis method, it can be applied not only to product quality control but also to pharmacokinetic studies such as bioavailability. In a pharmacokinetic study applying the method developed in this study, *p*-CA, an indicator substance of BC extract, was proven to be an effective analysis method because important parameters such as T_max_, C_max_, T_1/2_, and AUC_t_ were well-detected. This analysis method proved to be validated in accuracy, precision, and stability, so it is considered to be excellent in application to the analysis of indicator materials.

In this study, we used the UPLC-MS/MS method to detect *p*-CA extracted from BC in human plasma for pharmacokinetic studies. Specificity was evaluated by analyzing matrix blanks from six unique lots of matrix. We found that the lower limit of quantification (LLOQ) of *p*-CA in plasma was 0.2 ng/mL and the upper limit of quantification (ULOQ) was 20 ng/mL. The LLOQ is defined for chromatographic analysis as the lowest “acceptable” concentration used in routine calibration analysis, and the ULOQ is defined for chromatographic analysis as the highest “acceptable” concentration used in routine calibration analysis [14]. Therefore, concentrations which are below 0.2 ng/mL (LLOQ) should be mentioned as the zero concentrations. We showed the measured intraday, inter-day precision and accuracy for 0.2, 0.6, 8 and 16 ng/mL standard concentrations (Table 2). Since the inter-day and intra-day precision were both less than 15%, and the corresponding accuracy was 85 to 115%, it was suitable for the Acceptance criteria for accuracy and precision [14]. Moreover, the response was linear throughout the concentration range of the study, with the coefficient of determination (*r^2^*) of 0.999957. As the coefficient of determination is very close to 1, it can be seen that the regression equation is very suitable. The correlation equation was y = 6819.89x + 1013.91 in human plasma (Figure 4). Table 2 indicated that the main pharmacokinetic parameters of *p*-CA in human plasma after oral administration of BC. *p*-CA was absorbed rapidly and reached a maximum concentration and T_max_ of 21.95 ± 11.36 ng·mL^−1^ and 0.50 ± 0.35 h, respectively. The T_1/2_ of of *p*-CA was 0.9 ± 0.5 h. The AUC_t_ and AUC_inf_ of *p*-CA after oral administration of BC were 20.55 ± 1.50 ng·h·mL^−1^ and 20.82 ± 1.63 ng·h·mL^−1^, respectively. AUC_t_/AUC_inf_ was 98.7 ± 0.5%, which was found to be sufficient time to measure the parameters.

Children with growth delay compared with other kids in their class have often increased parental concern. GH therapy has been used as a resolution of these problems [1,2]. Many studies have demonstrated that GH and insulin-like growth factor-1 (IGF-1) have critical roles in bone homeostasis through increasing bone mass and linear bone growth [15,16,17]. GH stimulates the secretion of IGF-I, from the liver, then functions in an endocrine fashion [15,16]. GH also stimulates IGF-I locally in target tissues such as bone, acting in a paracrine or autocrine fashion [15,16]. IGF-1 has been proven to produce proliferation of MC3T3 osteoblasts and is an essential survival factor for numerous mammalian cell types [18], including osteoblasts [19]. However, children with GH treatment need long-term injection periods, creating unwanted side effects and parent burden with high cost. For this reason, many other alternative herbs are being studied.

One study reported increased longitudinal bone growth by aqueous extract of *Phyllostachyos Caulis*, which was able to support growth in adolescent rats via the upregulation of osteocalcin and IGF-1 levels [5]. Given the result of the previously referred study, BC extract (*p*-CA) was used, which is also derived from bamboo such as *Phyllostachyos Caulis* to investigate whether it is effective for height growth. Based on the pharmacokinetic data obtained above, we also performed the pilot study for pharmacodynamic study. In the pilot study, we evaluated effectiveness of *p*-CA extracted from BC on the growth of children’s height. Additionally, we also checked IGF-1 and Osteocalcin levels, which are important factors on growth, as well as height. The tested treatment (BC extract) in this study showed the effect of inducing height growth in children, but the degree was not statistically significant. On the other hand, the secretion of growth factors IGF-1 and osteocalcin, which are involved in height growth of young children, was increased. 

In this study, BC extracts showed a tendency to induce growth, so there was no statistical significance because there were not many participants in this study due to the characteristics of the pilot study. If the number of subjects to reach a degree of statistical significance are enrolled, it is judged that the significance will appear, and further research is necessary based on this. Moreover, the significant increase in changes in IGF-1 and osteocalcin, which are recognized as very important in height growth, shows the possibility of developing therapeutic agents in the future. The increase in IGF-1 and osteocalcin leads to height growth, but the 12-week study period is a bit shorter, so it is necessary to increase the study period to monitor height growth in future studies. This clinical study is recognized as a very useful study that collects basic data of the study as a study to determine the success of future clinical studies.

## 4. Conclusions

A sensitive and efficient LC-MS method was developed and validated for the determination of *p*-CA in human plasma, with a lower quantitation limit of 0.2 ng/mL. Validation experiments showed that the analysis showed excellent precision and accuracy over a range of concentrations. In this PK study, we were able to determine the dosage and concentration of *p*-CA in human plasma. In addition, through the pilot study, it was found that the treatment group increased in height compared to the placebo group. However, the *p*-value was not significant due to the small sample size, but statistically, when more than 98 clinical trials were conducted, a result that satisfies the *p*-value was derived. However, despite the small number of samples, IGF-1 and osteocalcin, which are helpful for bone growth, satisfied the *p*-value and were found to increase considerably in the treatment group.

Through this PK study and pilot study, it was possible to confirm the possibility that the extract in BC can be developed as a health functional food or medicine that can help height growth in the future.

## Figures and Tables

**Table 1:** Accuracy and precision for the analysis of *p*-Coumaric acid in human plasma.

Nominal (ng/mL)	Precision CV (%)		Accuracy (%)	
	Intra (*n* = 5)	Inter (*n* = 3)	Intra (*n* = 5)	Inter (*n* = 3)
0.2	5.6	6.4	103.8	108.4
0.6	2.3	2.4	100.0	100.9
8	1.2	2.1	99.0	99.6
16	1.0	1.3	99.2	101.3

**Table 2:** The main PK parameters of *p*-Coumaric acid in human plasma after oral administration of BC.

Parameter	Unit	Value (Mean ± SD)
C_max_	ng/mL	21.95 ± 11.36
AUC_t_	ng·h/mL	20.55 ± 1.50
AUC_inf_	ng·h/mL	20.82 ± 1.63
AUC_t_/AUC_inf_	%	98.7 ± 0.5
T_max_	h	0.50 ± 0.35
T_1/2_	h	0.9 ± 0.5

C_max_: Maximum plasma concentration, AUC_t_: area under the plasma concentration curve from administration to last observed, AUC_inf_: area under the plasma concentration curve extrapolated to infinite time, AUC_t_/AUC_inf_: area under the plasma concentration-time curve extrapolated from time t to infinity as a percentage of total AUC, T_max_: Time to maximum plasma concentration, Time until Cmax is reached, T_1/2_: plasma concentration half-life.

**Table 3:** Study participants’ baseline characteristics.

Variable	Group		*p*-Value
	Placebo	Treatment	
All participants	18	16	
Sex			
Male (n, %)	13 (72.2%)	9 (56.3%)	0.3307 ^1^
Female (n, %)	5 (27.8%)	7 (43.8%)	
Age			
Mean (SD)	9.00 (1.37)	7.94 (1.48)	0.0531 ^2^
6	1 (5.6%)	3 (18.8%)	
7	1 (5.6%)	5 (31.3%)	
8	5 (27.8%)	1 (6.3%)	
9	3 (16.7%)	4 (25.0%)	
10	6 (33.3%)	3 (18.8%)	
11	2 (11.1%)	0 (0.0%)	

SD: standard deviation. ^1^ *p*-values were derived from chi-square test. ^2^ *p*-values were derived from Fisher’s exact test.

**Table 4:** Efficacy Comparison by treatment group at each assessment periods.

Variable	Value		*p*-Value ^1^
	Placebo (*n* = 18)	Treatment (*n* = 16)	
**Height (cm)**	1.03 cm/3 month	1.43 cm/3 month	
Screening	126.93 ± 7.05	121.43 ± 7.89	0.0394 ^2^
Visit 1	127.38 ± 7.10	121.60 ± 7.98	0.0326 ^2^
Visit 2	127.88 ± 7.03	122.25 ± 8.18	0.0386 ^2^
Visit 3	128.41 ± 7.06	123.03 ± 7.84	0.0430 ^2^
Change from baseline	1.03 ± 0.51	1.43 ± 0.68	0.0639 ^2^
IGF-1	0.27	9.57	
Screening	69.85 ± 16.36	64.95 ± 20.85	0.3496 ^3^
Visit 3	70.12 ± 19.94	74.52 ± 19.38	0.3162 ^3^
Change from baseline	0.26 ± 13.69	9.57 ± 12.69	0.0488 ^2^
Osteocalcin			
Screening	66.80 ± 27.59	75.96 ± 30.49	0.3003 ^3^
Visit 3	58.72 ± 27.56	89.44 ± 19.92	0.0058 ^3^
Change from baseline	−8.08 ± 18.33	13.48 ± 25.12	0.0070 ^2^

^1^*p*-values were adjusted for observed value at baseline by using baseline value as covariate in analysis of covariance. Baseline value (screening or visit 1) was adjusted in ANCOVA analysis. ^2^*p*-values were derived from independent t-test. ^3^*p*-values were derived from Mann-Whitney’s U test. Shapiro-Wilk’s test was employed for test of normality assumption.

**Table 5:** Safety Comparison by Placebo and Treatment group at each assessment period.

Group				Group			
Variable	Placebo	Treatment	*p*-Value *	Variable	Placebo	Treatment	*p*-Value *
**SBP**				**WBC**			
Screening	100 ± 5	100 ± 7	0.4772	Screening	6.65 ± 1.03	7.41 ± 0.93	-
At week 12	100 ± 8	90 ± 8	0.2815	At week 12	6.79 ± 2.06	7.15 ± 2.24	0.4971
**DBP**				**Uric acid**			
Screening	65 ± 7	60 ± 8	0.2652	Screening	3.9 ± 0.6	4.1 ± 1.0	0.3695
At week 12	60 ± 10	60 ± 9	0.3423	At week 12	4.1 ± 1.0	3.8 ± 0.7	0.1371
**Pulse**				**Na**			
Screening	82 ± 8	84 ± 6	0.4238	Screening	140 ± 1	140 ± 1	0.3813
At week 12	78 ± 7	81 ± 5	0.1444	At week 12	141 ± 2	140 ± 2	0.2341
**K**				**HDL-C**			
Screening	4.2 ± 0.5	4.5 ± 0.4	-	Screening	66 ± 12	67 ± 12	0.4286
At week 12	4.3 ± 0.3	4.1 ± 0.5	0.4787	At week 12	62 ± 11	64 ± 14	0.4605
**Hemoglobin**				**LDL-C**			
Screening	12.8 ± 0.8	13.2 ± 0.4	0.4078	Screening	90 ± 18	108 ± 29	0.4286
At week 12	13.5 ± 0.8	13.3 ± 0.3	0.3671	At week 12	89 ± 21	115 ± 22	0.4605

SBP: systolic blood pressure, WBC: white blood cellm, DBP: diastolic blood pressure, K: potassium, Na: sodium, HDL-C: high density lipoprotein cholesterol, LDL-C: low density lipoprotein cholesterol. * *p*-values were adjusted for observed value at screening and week 12 by using screening values as the covariate in analysis of covariance and derived from independent t-test.

## Data Availability

Informed consent was obtained from all subjects involved in the study.
